# Olfactory bulbectomy-induced impairment of lipid utilization leads to abnormal glucose metabolism in mice

**DOI:** 10.1371/journal.pone.0333176

**Published:** 2025-09-29

**Authors:** Masanori Sugiyama, Hiroshi Tsuneki, Kengo Onishi, Koharu Yubune, Towa Yamagishi, Shota Sano, Yuki Tanaka, Ichiro Takasaki, Takeshi Sakurai, Masashi Yanagisawa, Keiichi Koizumi, Makoto Kadowaki, Shigeru Saito, Tsutomu Wada, Toshiyasu Sasaoka

**Affiliations:** 1 Department of Clinical Pharmacology, University of Toyama, Toyama, Japan; 2 Department of Integrative Pharmacology, University of Toyama, Toyama, Japan; 3 Department of Pharmacology, University of Toyama, Toyama, Japan; 4 Faculty of Medicine/WPI-IIIS, University of Tsukuba, Tsukuba, Japan; 5 Research Center for Pre-Disease Science, University of Toyama, Toyama, Japan; 6 Division of Presymptomatic Disease, Institute of Natural Medicine, University of Toyama, Toyama, Japan; University of Naples Federico II: Universita degli Studi di Napoli Federico II, ITALY

## Abstract

Increased lipid utilization after food odor perception is a recently identified Pavlovian cephalic phase response in fasted mice that serves to prevent diet-induced glucose intolerance. However, the impact of olfactory dysfunction on metabolic functions remains unclear. Since olfactory bulbectomized (OBX) rodents have been used as a model of irreversible complete anosmia, we investigated whether glucose, lipid, and energy metabolism change over time in OBX mice fed a normal chow diet (NCD) or high-fat diet (HFD). OBX caused constant hyperactivity and triphasic temporal changes in lipid and glucose metabolism. In the early stage, OBX disrupted the olfactory regulation of lipid utilization and reduced fasting serum fatty acid levels without affecting glucose tolerance. In the middle stage (10–40 weeks after OBX on NCD; 5–10 weeks after OBX on HFD), OBX mice showed a mild reduction in body weight gain and improved glucose tolerance. In the later stage, glucose tolerance did not improve in NCD-fed OBX mice, while glucose tolerance was impaired and the expression of hepatic genes related to lipid metabolism was abnormal in HFD-fed OBX mice. In the absence of orexin, which regulates the brain-liver network, HFD-fed OBX mice showed an improvement in, but not the subsequent impairment of glucose tolerance. These results suggest that OBX rapidly impairs lipid metabolism, which gradually exacerbates glucose metabolism, whereas the associated hyperactivity contributes to improvements in glucose metabolism. Therefore, the olfactory bulb plays an essential role in the maintenance of lipid and glucose homeostasis.

## Introduction

Classical Pavlovian conditioning refers to well-known physiological responses to food cues, such as the smell and sight of foods, during fasting in mammals, including humans [1]. The perception of food availability predictively promotes digestive and gastrointestinal absorptive functions before the ingestion of food. These predictive functions are also called ‘cephalic phase’ responses because they are controlled by the brain. The hypothalamus in the brain plays a major role in regulating the energy balance and metabolism of nutrients (e.g., glucose and lipids) [2]. We recently found that food odor perception during fasting acutely promoted lipid utilization via the hypothalamus and autonomic nervous system not only in the fasting state, but also in the subsequent refeeding state, and also prevented the development of glucose intolerance under a long-term high-fat diet (HFD)-fed condition in mice [3]. The functional link between food odor perception and lipid metabolism may be beneficial for maintaining metabolic homeostasis. However, the effects of a decreased sense of smell on metabolic functions remain unclear.

Although epidemiological evidence indicates that olfactory impairments are associated with obesity and type 2 diabetes, the causal relationship has yet to be elucidated [4,5]. Previous findings using genetically modified anosmic mice were inconsistent, i.e., detrimental or beneficial effects on body weight and the whole-body energy balance [6,7]. It is important to note that the anosmic mice used in previous studies may not have been suitable as a persistent olfactory dysfunction model because they showed only the partial loss of smell despite having some recovery abilities, including olfactory epithelium regeneration and/or olfactory neurogenesis [8]. Therefore, investigations using animals with profound and sustained olfactory impairments need to be conducted for a proper analysis.

The olfactory bulb is a central region that receives and integrates sensory information from the nose and serves as the starting point for olfactory perception in the brain. Olfactory bulbectomy provides irreversible complete anosmia in rodents [9]; however, metabolic changes have not yet been sufficiently characterized. Well-known changes in olfactory bulbectomized (OBX) rodents are hyperlocomotion and increased food intake [10–12]. Body weight and energy expenditure were previously shown to be unchanged or mildly reduced in OBX mice [6,12]. Early preliminary studies reported that olfactory bulbectomy improved glucose tolerance and reduced serum lipid levels in rats; however, the effects of confounding factors, such as body weight changes and age at bulbectomy, remain unclear [13–15]. Furthermore, the pharmacological and pharmacogenetic activation of the olfactory bulb reduced body weight gain and improved glucose tolerance under diet-induced obese conditions [7,16]. Therefore, the ultimate effects of olfactory bulbectomy on metabolic health have yet to be reported.

To clarify the role of the olfactory bulb in the regulation of metabolic functions, we herein examined time-dependent changes in glucose, lipid, and energy metabolism in OBX mice fed a normal chow diet (NCD) or HFD. The results obtained showed that olfactory bulbectomy caused triphasic temporal changes in lipid and glucose metabolism, and exacerbated HFD-induced glucose intolerance in the long term. The present study provides insights into diabetes prevention by targeting the olfactory system.

## Materials and methods

### Animals

Male mice were used in the present study. C57BL/6J mice were purchased from Japan SLC (Shizuoka, Japan). Orexin-deficient mice (*Orexin*^*-/-*^, the N11 generation backcrossed to C57BL/6J mice) were prepared as previously described [17]. Mice were maintained in a specific pathogen-free animal facility with a standard 12-h light:12-h dark cycle. Room lights were switched on at 7:00 [zeitgeber time (ZT) 0] and switched off at 19:00 (ZT12). Room temperature was controlled between 20 and 26^o^C. Mice were allowed free access to NCD (PicoLab Rodent Diet 20, PMI Nutrition International, USA) and water. In experiments under diet-induced obesity conditions, mice were fed 60 kcal% HFD (D12492, Research Diets, New Brunswick, NJ). Olfactory bulbectomy was performed under anesthesia as described below. Mice were sacrificed by cervical dislocation for tissue isolation. All experimental procedures used in this study were approved by the Committee of Animal Experiments at the University of Toyama (No. A2015PHA-25, A2018PHA-20, A2021PHA-12, A2024PHA-09), and all efforts were made to alleviate suffering in animal experiments.

### Food odor stimulation

A forced food-odor stimulation was performed with a FOS1 apparatus (i.e., a forced olfactory stimulation apparatus with 1 shading bottle) as previously described [3]. In brief, mice fasted for 24 h were placed in an airtight cage containing a shading bottle with holes. The bottle was filled with or without NCD. By sending compressed air into the bottle with an air pump (VP-6035, Techno Takatsuki, Osaka, Japan), mice were forcedly stimulated with the food odor released from the bottle.

### Olfactory bulbectomy

Olfactory bulbectomy was performed according to a standard method as previously described [18]. In brief, mice were anesthetized by an intraperitoneal (i.p.) injection of a mixture of medetomidine (0.75 mg/kg), midazolam (4 mg/kg), and butorphanol (5 mg/kg). The skull over the olfactory bulb was exposed by incising the scalp, and two holes with a diameter of 1.5 mm each were drilled. The olfactory bulbs were bilaterally removed by aspiration through the holes. Sham-operated mice were prepared by the same method except for the removal of the olfactory bulbs. Mice were allowed to recover for 7 days after surgery. At the end of each experiment, mice were sacrificed by cervical dislocation, and lesions of the olfactory bulbs were visually confirmed (S1 Raw images).

### Glucose and insulin tolerance tests

**Fig 1:**
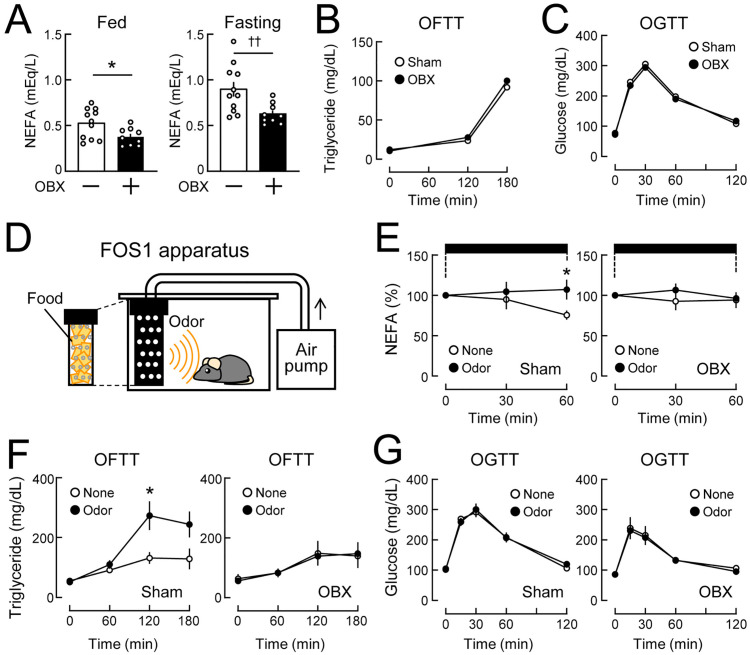
Early-stage changes in lipid metabolism in olfactory bulbectomized (OBX) mice fed a normal chow diet. C57BL/6J mice (7-8 weeks old) were subjected to olfactory bulbectomy, and lipid and glucose metabolism under a normal chow diet-fed condition were analyzed at indicated times. **(A)** Basal serum NEFA levels in OBX and sham-operated mice in the *ad libitum* fed (left) and 24-h fasting states (right). Experiments were conducted 4 (left) and 3 weeks (right) after bulbectomy. n = 9-11. **(B)** Serum triglyceride levels in the oral fat tolerance test (OFTT) in OBX and sham-operated (Sham) mice fasted for 24 **h.** Experiments were conducted 3 weeks after bulbectomy. n = 9-11. **(C)** Blood glucose levels in the oral glucose tolerance test (OGTT) in OBX and sham-operated mice fasted for 24 **h.** Experiments were conducted 2 weeks after bulbectomy. n = 10-11. **(D)** The FOS1 apparatus (the forced olfactory stimulation apparatus with 1 shading bottle). The shading bottle was filled with food pellets or was empty. **(E)** Changes in serum NEFA levels during the stimulation with the food odor or empty bottle in sham-operated (left) and OBX mice (right) fasted for 24 **h.** Experiments were conducted 2 (left) and 3 weeks (right) after bulbectomy. n = 5-6. Horizontal bars indicate the period of the olfactory stimulation for 1 **h. (F)** OFTT just after 1 h of the food odor stimulation in sham-operated (left) and OBX mice (right) fasted for 24 **h.** Experiments were conducted 2 weeks after bulbectomy. n = 5-7. **(G)** OGTT just after 1 h of the food odor stimulation in sham-operated (left) and OBX (right) mice fasted for 24 **h.** Experiments were conducted 4 weeks after bulbectomy. n = 3-6. Values are the means ± SEM. *P < 0.05 assessed by the Student’s *t*-*t*est. ^††^P < 0.01 assessed by the Welch’s *t*-tes*t*.

In oral glucose tolerance tests (Fig 1C and 1G), mice fasted for 24 or 25 h were orally administered glucose (2 g/kg). In i.p. glucose tolerance tests, mice fasted for 6 h were injected with glucose (2 g/kg, i.p.), except for Figs 3E, 3F, and 4E; glucose (1.5 g/kg) was i.p. injected into mice fasted for 6 h in Fig 3E and Fig 4E and into mice fasted for 16 h in Fig 3F. In insulin tolerance tests, mice fasted for 2 h were i.p. injected with insulin (Humulin R provided by Eli Lilly Japan, Kobe, Japan; 1 unit/kg). Blood samples were collected from the tail vein, and blood glucose levels were measured using a Statstrip XP3 glucose meter (11−110, Nipro, Osaka, Japan) with its sensors (11−106, Nipro) or a FreeStyle Freedom Lite glucose meter (70959−70, Abbott Japan, Tokyo, Japan) with its sensors (71409−70, Abbott Japan).

**Fig 2:**
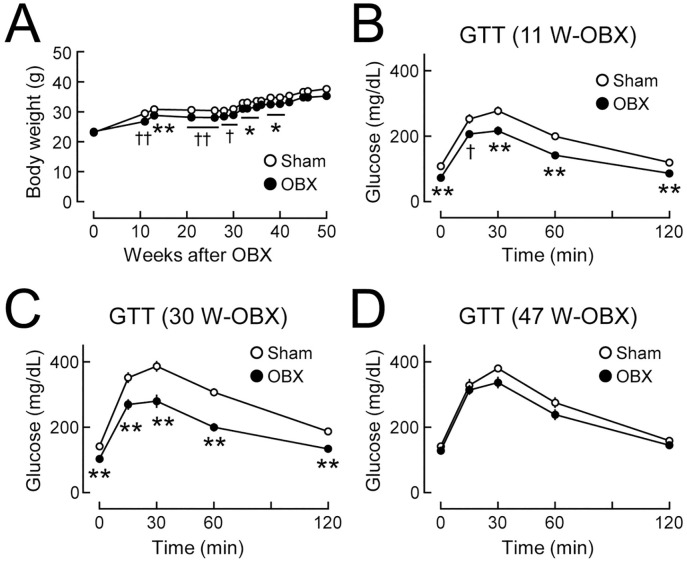
Temporal changes in glucose tolerance in olfactory bulbectomized (OBX) mice fed a normal chow diet. C57BL/6J mice (7-8 weeks old) underwent olfactory bulbectomy, and body weight and glucose metabolism under a normal chow diet-fed condition were compared between OBX and sham-operated (Sham) mice. **(A)** Body weights in OBX and sham-operated mice. n = 15-16. **(B-D)** Glucose tolerance tests (GTT) conducted 11 weeks **(B)**, 30 weeks **(C)**, and 47 weeks (D) after bulbectomy. n = 15-16. Values are the means ± SEM. *P < 0.05, **P < 0.01 assessed by the Student’s *t*-tes*t*. ^†^P < 0.05, ^††^P < 0.01 assessed by the Welch’s *t*-test.

### Analysis of serum parameters

Serum insulin levels in glucose (2 g/kg, i.p.)-stimulated insulin secretion tests after 6 h of fasting were measured with a Mouse/Rat insulin ELISA kit (M1108, Morinaga Institute of Biological Science, Kanagawa, Japan). Serum non-esterified fatty acid (NEFA) levels were measured with the LabAssay NEFA kit (633–52001 or 299–94301, Wako Pure Chemical, Osaka, Japan). In the oral fat tolerance test, C57BL/6J mice fasted for 24–25 h were orally administered olive oil (300 µL), and their serum triglyceride levels were measured with the LabAssay Triglyceride kit (632–50991 or 291–94501, Wako). All experiments were performed using a microplate leader (FilterMax F5 or SpectraMax i3, Molecular Devices Japan, Tokyo, Japan), according to the manufacturer’s instructions.

### *In vivo* analysis of energy metabolism

Mice were acclimated to the metabolic cages of a small animal’s metabolism-measuring system (MK-5000RQ, Muromachi Kikai, Tokyo, Japan) for at least 12 h, and locomotor activity, food intake, O_2_ consumption, CO_2_ production, energy expenditure, and the respiratory quotient (RQ) were then simultaneously measured for 24 h. In experiments to measure food intake during refeeding after fasting (Fig 5I, Fig 5L, and S4 Fig), mice fasted for 6 h (ZT2-ZT8) were fed NCD, and cumulative food intake in each mouse was measured using food containers (Roden Cafe, Oriental Yeast, Tokyo, Japan) 1, 2, 3, and 6 h after refeeding. Locomotor activity after a cage change (Fig 5H, Fig 5K, and S4 Fig) was measured using a video tracking system (Any-maze, ver. 4.60, Stoelting, IL, USA) as an exploratory behavior in a novel environment. To investigate the profiles of food intake and locomotor activity under a thermoneutral condition (Fig 5K and 5L), mice were maintained in a thermo-controlled chamber (HC-10, Shinfactory, Fukuoka, Japan) at 30^o^C for 7 days. Body temperature was measured from the rectum using a microprobe thermometer (BAT-12, Muromachi Kikai) with T-probe (RET-2, Muromachi Kikai).

**Fig 3:**
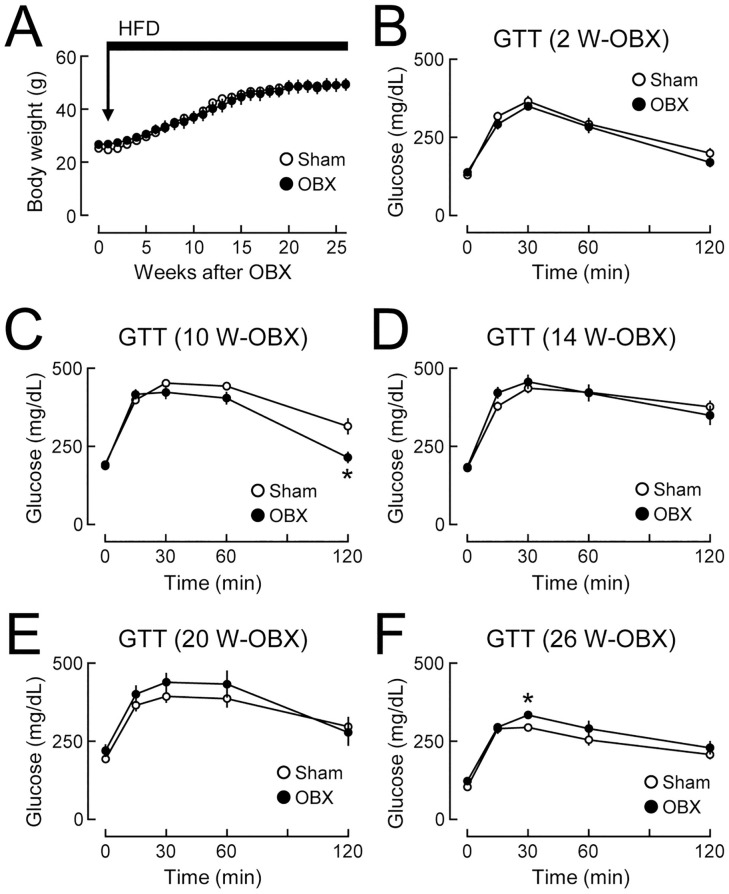
Temporal bidirectional changes in glucose tolerance in olfactory bulbectomized (OBX) mice fed a high-fat diet (HFD). C57BL/6J mice (10 weeks old) were subjected to olfactory bulbectomy and HFD feeding began 1 week later. **(A)** Body weights in OBX and sham-operated (Sham) mice. n = 6-7. The horizontal bar indicates the period of HFD feeding. **(B-F)** Glucose tolerance tests (GTT) conducted 2 weeks **(B)**, 10 weeks **(C)**, 14 weeks **(D)**, 20 weeks **(E)**, and 26 weeks (F) after olfactory bulbectomy. n = 6-7. Values are the means ± SEM. *P < 0.05, assessed by the Student’s *t*-*t*est.

**Fig 4:**
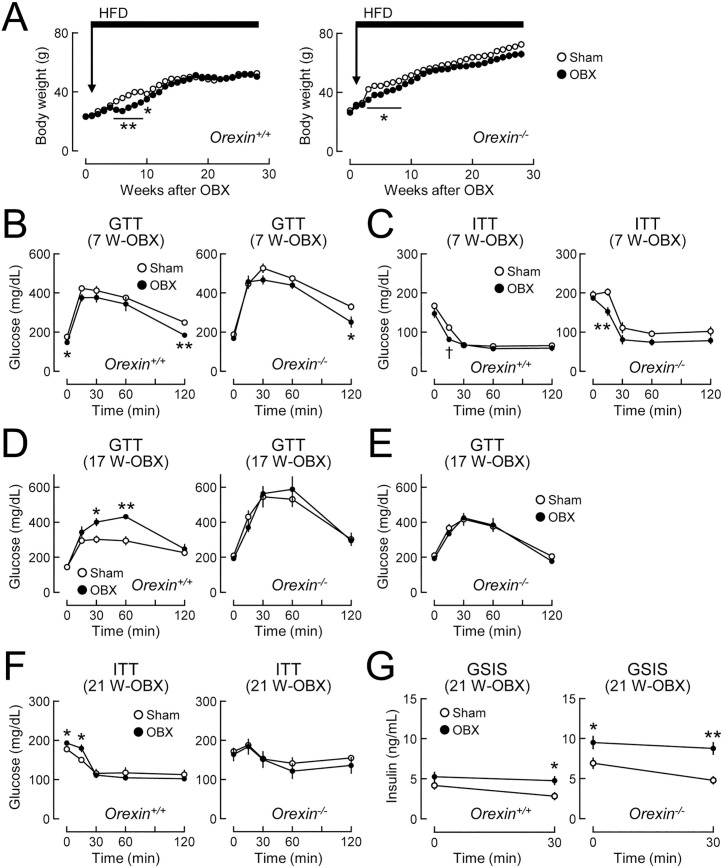
Different dependencies on orexin of bidirectional changes in glucose tolerance in olfactory bulbectomized (OBX) mice fed HFD. *Orexin*^*+/+*^ and *Orexin*^*-/-*^ mice (8-10 weeks old) were subjected to OBX and HFD feeding began 1 week later. **(A)** Body weights in *Orexin*^*+/+*^ (left) and *Orexin*^*-/-*^ (right) mice after OBX or sham operation (Sham). n = 5-7. Horizontal bars indicate the period of HFD feeding. **(B-C)** Glucose tolerance tests (B, GTT) and insulin tolerance tests (C, ITT) in *Orexin*^*+/+*^ mice (left) and *Orexin*^*-/-*^ (right) mice fed HFD for 6 weeks (i.e., 7 weeks after surgery, 7 W-OBX). n = 5-7. **(D)** GTT in *Orexin*^*+/+*^ mice (left) and *Orexin*^*-/-*^ (right) mice fed HFD for 16 weeks (17 W-OBX). n = 5-7. **(E)** GTT in *Orexin*^*-/-*^ mice fed HFD for 16 weeks (17 W-OBX) using a lower dose of glucose (1.5 g/kg, i.p.) than in panel D (2 g glucose/kg, i.p.). n = 6/group. **(F-G)** ITT (F) and glucose-stimulated insulin secretion tests (G) in *Orexin*^*+/+*^ mice (left) and *Orexin*^*-/-*^ (right) mice fed HFD for 20 weeks (21 W-OBX). n = 5-7. Values are the means ± SEM. *P < 0.05, **P < 0.01 assessed by the Student’s *t*-tes*t*. ^†^P < 0.05 assessed by the Welch’s *t*-test.

**Fig 5:**
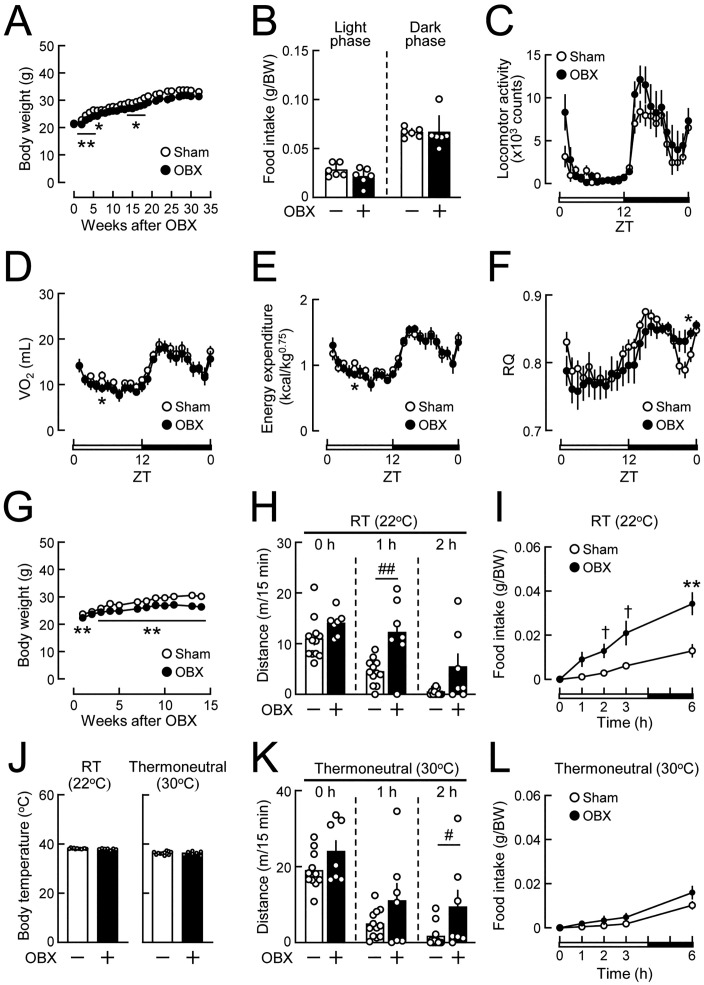
Effects of olfactory bulbectomy on food intake, locomotor activity, and energy metabolism. C57BL/6J mice (7 weeks old) were subjected to olfactory bulbectomy and maintained on NCD. **(A)** Body weights in olfactory bulbectomized (OBX) and sham-operated (Sham) mice in panels B-F. n = 6/group. **(B)** Food intake during the light and dark phases, measured 11 weeks after OBX. n = 6/group. **(C-F)** Locomotor activity **(C)**, oxygen consumption **(D)**, energy expenditure **(E)**, and the respiratory quotient (F) measured 15 weeks after surgery. n = 6/group. **(G)** Body weights in OBX and sham-operated mice in panels H-L. n = 7-12. **(H)** Locomotor activity (total distance) measured 0 h (0-15 min), 1 h (60-75 min), and 2 h (120-135 min) after a cage change at room temperature (RT, 22^o^C). Experiments were conducted 10 weeks after surgery. n = 7-12. **(I)** Cumulative food intake after 6 h of fasting at RT (22^o^C). Experiments were conducted 13 weeks after surgery. n = 7-12. **(J)** Body temperatures in OBX and sham-operated mice that were maintained at RT (22^o^C, left) or at a thermoneutral temperature (30^o^C, right) for 1 week. n = 7-12. **(K)** Locomotor activity after a cage change at 30^o^C. Experiments were conducted 15 weeks after surgery. n = 7-12. **(L)** Cumulative food intake after 6 h of fasting at 30^o^C. Experiments were conducted 15 weeks after surgery. n = 7-12. Values are the means ± SEM. *P < 0.05, **P < 0.01 assessed by the Student’s *t*-tes*t*. ^†^P < 0.05 assessed by the Welch’s *t*-test. ^#^P < 0.05, ^##^P < 0.01 assessed by the Mann-Whitney U-test.

### Microarray assay and pathway analysis

OBX and sham-operated control mice were fed NCD or HFD for 25 weeks. The liver was then isolated, snap-frozen with liquid nitrogen, and stored at -80^o^C until used. RNA extraction and a microarray analysis using a GeneChip system with the GeneChip Mouse Gene 2.0 ST Array (Affymetrix, CA, USA) were performed as previously described [17,19]. Differentially expressed genes (fold-change >1.2; P < 0.05, n = 5/group) were identified by GeneSpring software (Agilent Technologies, CA, USA), and their relevant pathways were examined using Ingenuity Pathway Analysis software (IPA, Qiagen, Venlo, the Netherlands). To identify pre-disease signals in microarray data, a dynamical network biomarker (DNB) analysis was performed according to a previously described method [20].

### Reverse transcription-quantitative polymerase chain reaction (PCR)

The liver tissues isolated from OBX and sham-operated control mice fed HFD for 25 weeks were immersed in RNAprotect Tissue Reagent (76106, Qiagen) at 4^o^C overnight, frozen with liquid nitrogen, and then stored at −80°C until used. Total RNA was extracted with TRIsure reagent (BIO-38032, Nippon Genetics, Tokyo, Japan) and subjected to reverse transcription using the ReverTra Ace qPCR RT Master Mix with the gDNA Remover (FSQ-301, Toyobo, Osaka, Japan). Quantitative PCR (40 cycles at 95^o^C for 10 sec, 62^o^C for 20 sec, and 72^o^C for 15 sec) was conducted using the Brilliant III Ultra-Fast SYBR Green qPCR Master Mix (600882, Agilent Technologies) and Mx3000p/3005p qPCR system (Stratagene, Tokyo, Japan). These procedures were performed in accordance with the manufacturers’ instructions. The relative expression level was evaluated by calculating the ratio of target mRNA to an internal control (*18S* rRNA) in each sample. The PCR primers used were as follows: *Vsig4*; 5’-GACTTGACCACTAATGGGACTGGAA-3’ for the forward primer, 5’-GTCCTGCAGCGGAACAAGATATAA-3’ for the reverse primer, *Onecut1*; 5’-TTCCAGCGCATGTCGGCGCTC-3’ for the forward primer, 5’-GGTACTAGTCCGTGGTTCTTC-3’ for the reverse primer, and *18S* rRNA; 57-GTAACCCGTTGAACCCCATT-3’ for the forward primer, 5’-CCATCCAATCGGTAGTAGCG-3’ for the reverse primer.

### Statistical analysis

All data are expressed as the mean ± standard error of the mean (SEM). In comparison of two groups, the F-test was first performed to assess the equality of two population variances. When the population variances are equal, significant differences between the means of two groups were assessed by the unpaired two-tailed Student’s *t*-test. When the population variances are unequal, the Welch’s *t*-test was used to compare two groups. On the other hand, in experiments where the population distribution was not assumed to be normal (i.e., in Fig 5K, Fig 5H, and S4 Fig where locomotor activity in a control group decreased to undetectable levels due to the time-dependent decrease in novelty during the 2-hour measurement period), the Mann-Whitney U-test was used to evaluate differences between two groups. P < 0.05 was considered to be significant.

## Results

### Olfactory bulbectomy rapidly impairs lipid metabolism

To investigate whether the olfactory bulb contributes to the regulation of lipid and glucose metabolism, metabolic functions were compared between OBX and sham-operated control mice 2, 3, or 4 weeks after surgery. OBX mice showed lower serum levels of NEFA than sham-operated mice in the *ad libitum* fed and 24-h fasting states (Fig 1A). Serum triglyceride levels in the oral fat tolerance test (Fig 1B) and blood glucose levels during the glucose tolerance test (Fig 1C) were similar between OBX and sham-operated control mice. Therefore, lipid mobilization during fasting was selectively impaired in the early stage of OBX. To further examine the early-stage effects of olfactory bulbectomy on the food odor-induced promotion of lipid utilization, OBX and sham-operated mice were exposed to a NCD odor using the FOS1 apparatus in which mice constantly received the food odor (Fig 1D). The food odor stimulation increased serum NEFA levels during fasting in sham-operated mice, which is consistent with our previous findings [3]; however, this effect was not observed in OBX mice (Fig 1E). The food odor stimulation increased serum triglyceride levels during OFTT followed by odor stimuli in sham-operated mice, while this effect disappeared in OBX mice (Fig 1F). The food odor stimulation did not affect blood glucose levels during GTT (Fig 1G). These results suggest that olfactory bulbectomy rapidly disrupted the olfactory regulation of lipid metabolism without affecting glucose metabolism.

### Olfactory bulbectomy causes late-onset bidirectional changes in glucose tolerance

To investigate whether metabolic functions changed over time after olfactory bulbectomy, the profiles of lipid and glucose metabolism were compared between OBX and sham-operated control mice fed NCD in the long term. Body weight gain was slightly reduced 10–40 weeks (i.e., the middle stage) after olfactory bulbectomy, whereas no significant difference in body weight was observed between OBX and sham-operated control mice in the later stage (Fig 2A). Glucose tolerance improved 11 and 30 weeks after olfactory bulbectomy (in the middle stage; Fig 2B and 2C), whereas no significant difference in glucose tolerance was observed 47 weeks after olfactory bulbectomy (in the late stage; Fig 2D). Another group of OBX mice showed slight reductions in body weight gain (S1 Fig). Under this condition, OBX mice showed improved glucose tolerance only 20 weeks after olfactory bulbectomy (in the middle stage; S1 Fig). Temporal improvements in glucose tolerance were more apparent in OBX mice with weight loss than in those without weight loss (Fig 2, S1 Fig).

To investigate whether olfactory bulbectomy also affected glucose tolerance under diet-induced obese conditions, time-dependent changes in glucose tolerance were analyzed in OBX and sham-operated mice fed HFD. In this analysis, body weight gain was similar between OBX and sham-operated mice (Fig 3A). Glucose tolerance was not affected after 2 weeks (Fig 3B) and improved 10 weeks after OBX (Fig 3C). The improvement in glucose tolerance disappeared 14 weeks after OBX (Fig 3D). Glucose tolerance was slightly exacerbated 20 weeks after OBX (Fig 3E) and was significantly exacerbated 26 weeks after OBX (Fig 3F). These results demonstrate that olfactory bulbectomy caused bidirectional changes in glucose tolerance under HFD-fed conditions, similarly to, but more rapidly than those under NCD-fed conditions.

To elucidate the mechanisms underlying the biphasic changes in glucose tolerance in OBX mice fed HFD, the functional relationship between olfactory bulbectomy-induced metabolic changes and orexin, a key factor for the central regulation of glucose metabolism [21,22], was investigated using *Orexin*^*-/-*^ and wild-type *Orexin*^*+/+*^ mice fed HFD. In this analysis, body weight gain was temporarily reduced 5–10 weeks after OBX in both *Orexin*^*+/+*^ and *Orexin*^*-/-*^ mice fed HFD (Fig 4A). Glucose tolerance and insulin sensitivity improved 7 weeks after OBX in both *Orexin*^*+/+*^ and *Orexin*^*-/-*^ mice fed HFD (Fig 4B and 4C). However, 17 weeks after OBX, glucose tolerance was exacerbated in *Orexin*^*+/+*^ mice fed HFD (Fig 4D). Glucose tolerance was markedly impaired by orexin deficiency *per se*, as previously reported [22]. OBX did not exacerbate glucose tolerance in *Orexin*^*-/-*^ mice fed HFD at this time point (Fig 4D). Similar results were obtained when a lower dose (1.5 g/kg) of glucose was administered in the glucose tolerance test to avoid the possible saturation of blood glucose elevations in OBX- and sham-operated-*Orexin*^*-/-*^ mice fed HFD (Fig 4E). Furthermore, insulin sensitivity was slightly reduced 21 weeks after OBX in *Orexin*^*+/+*^ mice fed HFD, whereas insulin resistance in *Orexin*^*-/-*^ mice fed HFD was not exacerbated by OBX (Fig 4F). Serum insulin levels in glucose-stimulated insulin secretion tests increased 21 weeks after OBX in both *Orexin*^*+/+*^ and *Orexin*^*-/-*^ mice fed HFD (Fig 4G). No changes in food intake, locomotor activity, energy expenditure, or RQ were observed 25 weeks after OBX in *Orexin*^*+/+*^ and *Orexin*^*-/-*^ mice fed HFD (S2 Fig). These orexin-dependent/independent profiles indicate that the mechanisms for olfactory bulbectomy-induced improvements differed from those for the subsequent impairment in glucose tolerance.

### Mechanisms underlying bidirectional changes in glucose tolerance following olfactory bulbectomy

To elucidate the mechanisms underlying improved glucose tolerance in the middle stage of OBX, the state of the energy balance was analyzed in OBX mice fed NCD. Body weights were slightly lower in OBX mice than in sham-operated mice in the middle stage (Fig 5A). OBX did not affect food intake in the light or dark phase under *ad libitum* fed conditions (Fig 5B). In the metabolic cage analysis after acclimation, OBX did not significantly affect locomotor activity, oxygen consumption, or energy expenditure, while RQ in the dark phase increased in OBX mice (Fig 5C-5F). We then investigated the effects of the environment on locomotor activity and food intake in OBX mice with weight loss during the middle stage (Fig 5G). When exposed to a novel environment provided by a cage change, locomotor activity at 1 and 2 h was higher in OBX mice than in sham-operated mice (Fig 5H). When mice that were fasted for 24 h were refed for 6 h, cumulative food intake was markedly higher in OBX mice than in sham-operated mice (Fig 5I). Similar increases in locomotor activity and food intake were observed 48 weeks after OBX (S4 Fig). To further investigate the effects of changes in the ambient temperature, mice that were maintained at the housing room temperature (22^o^C) were acclimated to a thermoneutral temperature (30^o^C) for 1 week. Body temperatures did not change under these conditions in OBX or sham-operated mice (Fig 5J). Increased locomotor activity in OBX mice was observed at 30^o^C (Fig 5K), similar to the responses observed at 22^o^C (Fig 5H). On the other hand, no significant difference was noted in cumulative food intake during 6 h of refeeding after fasting between OBX and sham-operated mice at 30^o^C (Fig 5L), unlike the responses observed at 22^o^C (Fig 5I). These results indicate that increases in locomotor activity and food intake, but not basal energy expenditure, affected the body weights of OBX mice.

**Fig 6:**
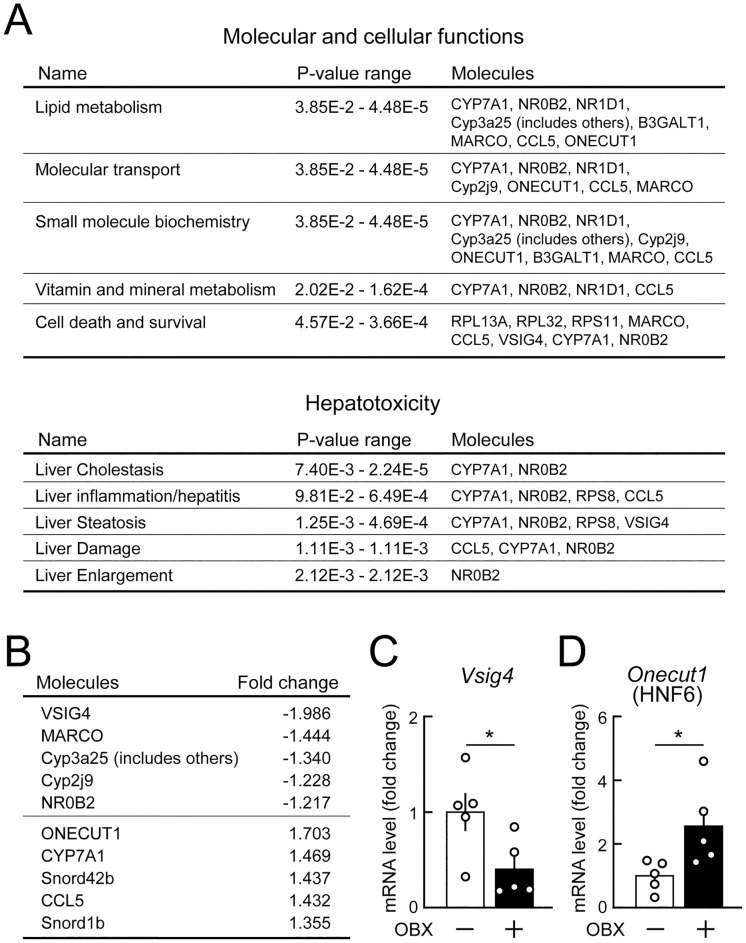
GeneChip analysis of hepatic gene expression in olfactory bulbectomized (OBX) mice chronically fed HFD. C57BL/6J mice (10 weeks old) were subjected to OBX, and HFD feeding began 1 week later. The liver was isolated 25 weeks after HFD feeding (i.e., 26 weeks after olfactory bulbectomy). **(A-B)** GeneChip/IPA analysis of hepatic gene expression in OBX and sham-operated mice fed HFD. **(A)** Pathway related to changes in hepatic gene expression in OBX mice in the category of Molecular and cellular functions and Hepatotoxicity. Molecules mapped to each biological pathway are shown using gene symbols. n = 5/group. **(B)** The most highly up- or down-regulated genes in the liver of OBX mice. n = 5/group. **(C-D)** A reverse transcription-quantitative PCR analysis of the expression levels of the *Vsig4* (C) and *Onecut1* genes (D) in the livers of OBX mice fed HFD. n = 5/group. Values are the means ± SEM. *P < 0.05 assessed by the Student’s *t*-*t*est.

To investigate the mechanisms underlying metabolic abnormalities in the late stage of OBX, we analyzed hepatic gene expression in OBX and sham-operated mice fed HFD for 25 weeks because we previously reported that a food odor stimulation enhanced metabolic activity in the liver [3]. No significant differences were observed in body or tissue weights between OBX and sham-operated mice at any indicated time point, except for temporal reductions in inguinal and epididymal white adipose tissue weights 2 weeks after OBX (S3 Fig). A GeneChip/IPA analysis of hepatic gene expression profiles in OBX and sham-operated mice fed HFD showed that olfactory bulbectomy-induced changes were related to the pathway of “lipid metabolism” in the category of Molecular and cellular functions and “liver steatosis” in the category of Hepatotoxicity (Fig 6A). The most highly down- and up-regulated genes were *Vsig4* (encoding for V-set and immunoglobulin domain containing 4 protein) and *Onecut1* (encoding for hepatocyte nuclear factor 6 protein, the HNF6 protein), respectively, in the liver of OBX mice fed HFD (Fig 6B). Significant changes in the expression of these hepatic genes were also detected by the quantitative PCR analysis (Fig 6C and 6D). The DNB analysis showed that hepatic gene expression profiles in OBX mice fed HFD were related to the transition states of metabolic processes, including glucose transport and carbohydrate metabolism (S5 Fig). We also analyzed hepatic gene expression profiles in OBX and sham-operated mice maintained on NCD for 26 weeks. The GeneChip/IPA analysis identified “lipid metabolism” in the category of Molecular and cellular functions and “liver steatosis” in the category of Hepatotoxicity as pathways related to expression profiles in OBX mice fed NCD (S6 Fig), similar to those fed HFD, as shown above (Fig 6A). Therefore, the chronic OBX deranged the expression of hepatic genes responsible for metabolic functions under both NCD- and HFD-fed conditions.

## Discussion

The present results revealed triphasic temporal changes in lipid and glucose metabolism in OBX mice. In the early stage after OBX, fasting serum NEFA levels were reduced and food odor stimulation-induced lipid utilization disappeared, whereas glucose metabolism remained unchanged. In the middle stage (approximately 10 weeks after olfactory bulbectomy), OBX mice showed hyperactivity and hyperphagia, resulting in a mild reduction in body weight gain in association with improved glucose tolerance. In the later stage (>40 weeks after OBX on NCD; > 15 weeks after OBX on HFD), the improvement in glucose tolerance disappeared and it actually worsened, particularly under HFD-fed conditions independently of body weight. Therefore, the removal of the olfactory bulb primarily impaired lipid utilization and secondarily resulted in the dysregulation of glucose metabolism.

Food perception during fasting elicits Pavlovian conditioning. As part of its physiological functions, a food odor stimulation during fasting increases serum NEFA levels before food ingestion and promotes lipid absorption and utilization during refeeding [3]. In this process, olfactory memory recall in the piriform cortex causes biphasic changes in the balance of hypothalamic agouti-related peptide and proopiomelanocortin neuronal activities and the autonomic nerve balance, thereby leading to lipid mobilization from white adipose tissue in the fasting state and gut lipid absorption and hepatic lipid utilization in the refeeding state [3]. The present study demonstrated that these effects of the food odor stimulation were eliminated in OBX mice, suggesting that the activation of the olfactory bulb is an essential initial step in the central mechanism linking olfaction and lipid utilization.

The functional link between olfaction and lipid metabolism is not only caused by food odors, but also by some non-food odors. For example, the scent of grapefruit oil was previously shown to increase plasma NEFA and glycerol levels via adipose tissue lipolysis by activating the sympathetic nervous system [23,24]. Furthermore, muscone, an attractive odorant for mice, activated specific neurons in the olfactory bulb [25] and increased serum NEFA levels [3]. The continuous pharmacological activation of major output neurons in the olfactory bulb were found to reduce RQ in diet-induced obese mice, indicating that the strong activation of the olfactory bulb caused energy substrate switching from glucose to lipid utilization [16]. As a counter phenomenon, we showed that serum NEFA levels decreased in the early stage of OBX. Increases in RQ (i.e., energy substrate switching from lipid to glucose utilization) were observed in the middle stage of OBX. Collectively, these results suggest that OBX persistently impaired the regulation of lipid metabolism, including adipose tissue lipolysis and whole-body lipid utilization, possibly by deranging the autonomic regulation of peripheral metabolic functions.

Olfactory bulbectomy-induced hyperactivity and hyperphagia [12] have opposing effects on the energy balance. Since OBX mice showed no changes in body temperature or energy expenditure, the relative contributions of hyperactivity and hyperphagia appear to affect the energy balance. Olfactory bulbectomy-induced hyperphagia has been associated with the increased expression of neuropeptide Y, an orexigenic factor, in the hypothalamus [11]; however, the underlying mechanisms for hyperactivity in OBX rodents remain unknown [9]. We herein demonstrated that the increase in food intake by OBX mice, which was observed at room temperature, disappeared under a thermoneutral condition, whereas locomotor activity was insensitive to the ambient temperature. Body weight gain remained unchanged or was mildly reduced in the middle stage of OBX, as previously reported [6,12], implying that increased locomotor activity predominantly affected the body weights of OBX mice under the present rearing environment. Greater reductions in body weight gain were associated with more apparent improvements in glucose tolerance in OBX mice (Fig 2, S1 Fig). These results suggest that weight loss due to increased locomotor activity improved glucose tolerance in the middle stage of OBX. Glucose tolerance mildly improved, even in a group of OBX mice with no significant weight loss (S1 Fig), which suggests that a mechanism independent of body weight also promoted glucose metabolism in OBX mice. Physical exercise enhances glucose uptake in skeletal muscles and promotes glycemic control [26]. More detailed studies on changes in muscle metabolic functions in OBX mice will provide further insights.

In contrast to the middle stage, OBX did not improve glucose tolerance under the NCD-fed condition and exacerbated it under the HFD-fed condition. *Orexin*^*-/-*^ mice were used to characterize time-dependent changes in glucose tolerance after OBX because they are the only known mouse model that can be used to modulate the central regulation of glucose metabolism without affecting the functional link between olfaction and lipid utilization [3,21,22]. Orexin regulates the brain-liver network through the autonomic nervous system to prevent hepatic insulin resistance and metabolic dysfunction-associated steatohepatitis [21,22]. Regarding the opposite changes in glucose tolerance in OBX mice in the middle and late stages, only the latter depended on orexin, which suggests that these changes were caused by independent mechanisms. In our GeneChip analysis, OBX induced the abnormal expression of hepatic genes related to hepatic toxicity and lipid metabolism in the late stage under the HFD-fed condition. Decreases in the expression of *Vsig4* and increases in that of *Hnf6*, as observed in the liver of OBX mice, are relevant to glucose intolerance and hepatic steatosis [27,28]. These findings suggest that OBX exacerbated glucose tolerance by disrupting the brain-liver network in the late stage. In the present study, dysregulated lipid metabolism was observed during all stages after OBX (e.g., reduced serum NEFA levels in the early stage, reduced lipid utilization in the middle stage, and abnormal hepatic gene expression related to lipid metabolism in the late stage). Therefore, OBX may persistently impair lipid utilization, leading to the gradual deterioration of hepatic metabolic functions, which manifested in the late phase. In parallel, hyperactivity in OBX mice may have continuously promoted glucose metabolism and induced temporal improvements in glucose tolerance, as observed in the middle stage. The results showing triphasic changes in metabolic functions in OBX mice may reconcile previous discrepancies (e.g., improvements in glucose tolerance by decreased [6] and increased olfactory activity [7]).

The present study had a number of limitations that need to be addressed. OBX rodents have been widely used as an animal model of depression and other mental disorders [9]. However, they have recently been reevaluated because OBX mice exhibit hyperactivity, decreased aggression, and a diminished sucrose preference, all of which cannot be explained by depression and anxiety, but rather by anosmia [9]. Therefore, we suggest that the changes observed in glucose and lipid metabolism in OBX mice were mainly due to the loss of smell. In addition, since we investigated metabolic changes in mice only, it remains unknown whether similar changes in lipid and glucose metabolism are caused by olfactory bulb dysfunctions in humans. The relationship between olfaction and the metabolic status is very complex [6]. Olfactory dysfunctions in humans have been associated with obesity, insulin resistance, and diabetes [4]. Nevertheless, obese human subjects showed increased olfactory sensitivity and enhanced brain responses to the odor of energy-dense food (i.e., chocolate), than energy-poor food (i.e., cucumber) [29,30]. A recent study reported that an odor imagery ability was associated with an increased body mass index [31], whereas another study found no effects of obesity or the body mass index on brain responses to food odors [32]. Therefore, further studies are needed to clarify the mechanisms by which olfactory dysfunctions of different severities and durations affect metabolic functions in humans.

## Conclusions

The present study demonstrated that the olfactory bulb plays an essential role in the maintenance of metabolic homeostasis in mice because OBX rapidly impaired lipid metabolism and gradually exacerbated glucose metabolism, particularly under diet-induced obesity conditions. Therefore, the olfactory system may be an important target for preventing the development of metabolic disorders, including obesity and type 2 diabetes.

## Supporting information

S1 Fig Body weight-independent temporal changes in glucose tolerance in olfactory bulbectomized (OBX) mice fed a normal chow diet (NCD). C57BL/6J mice (7 weeks old) were subjected to OBX and maintained on NCD. (A) Similar body weight gain in OBX and sham-operated (Sham) mice. n = 5–6. (B-F) Glucose tolerance tests (GTT) conducted 2 weeks (B), 6 weeks (C), 10 weeks (D), 20 weeks (E), and 32 weeks (F) after OBX. n = 5–6. Values are the means ± SEM. *P < 0.05 assessed by the Student’s *t*-test.
(TIF)

S2 Fig No effect of olfactory bulbectomy on food intake, locomotor activity, or energy metabolism in wild-type or orexin-deficient mice fed a high-fat diet (HFD). *Orexin*^*+/+*^ and *Orexin*^*-/-*^ mice (8–10 weeks old) underwent olfactory bulbectomy, and HFD feeding began 1 week later. A metabolic cage analysis was conducted on mice fed HFD for 24 weeks (i.e., 25 weeks after surgery). (A-B) Food intake in *Orexin*^*+/+*^ mice (A) and *Orexin*^*-/-*^ mice (B). n = 5–6. (C-D) Locomotor activity in *Orexin*^*+/+*^ mice (C) and *Orexin*^*-/-*^ mice (D). n = 5–6. (E-F) Energy expenditure in *Orexin*^*+/+*^ mice (E) and *Orexin*^*-/-*^ mice (F). n = 5–6. (G-H) The respiratory quotient in *Orexin*^*+/+*^ mice (G) and *Orexin*^*-/-*^ mice (H). n = 5–6. Values are the means ± SEM. The significance of differences was evaluated by the Student’s *t*-test. The mice used were the same as those in Fig 4.
(TIF)

S3 Fig Effects of olfactory bulbectomy on body and tissue weights in olfactory bulbectomized (OBX) mice fed a high-fat diet (HFD). C57BL/6J mice (10 weeks old) underwent olfactory bulbectomy, and HFD feeding began 1 week later. Body weights and weights of peripheral tissues [the liver, inguinal white adipose tissue (iWAT), and epididymal white adipose tissue (eWAT)] in OBX and sham-operated mice sacrificed by cervical dislocation 1 week (A-B), 2 weeks (C-D), 3 weeks (E-F), 10 weeks (G-H), and 26 weeks (I-J) after surgery. n = 4–5/group. The mice used in panels I-J were the same as those in Fig 6. Data are presented as mean ± SEM. *P < 0.05, **P < 0.01 assessed by the Student’s *t*-test.
(TIF)

S4 Fig Increases in locomotor activity and food intake in mice that were persistently maintained on a normal chow diet (NCD). (A) Locomotor activity (total distance) in olfactory bulbectomized (OBX) and sham-operated mice, measured 0 h (0–15 min), 1 h (60–75 min), and 2 h (120–135 min) after a cage change at room temperature (RT, 22^o^C). Experiments were conducted 49 weeks after surgery. n = 15–16. (B) Cumulative food intake after 6 h of fasting in OBX and sham-operated mice at RT (22^o^C). Experiments were conducted 48 weeks after surgery. n = 15–16. Values are the means ± SEM. *P < 0.05 assessed by the Student’s *t*-test. ^†^P < 0.05 assessed by the Welch’s *t*-test. ^##^P < 0.01 assessed by the Mann-Whitney U-test. The mice used were the same as those in Fig 2.
(TIF)

S5 Fig Profiles of dynamical network biomarkers identified in the liver of olfactory bulbectomized (OBX) mice fed a high-fat diet (HFD). Profiles of hepatic gene expression in OBX mice fed HFD for 25 weeks relative to those in sham-operated mice, as investigated by a dynamical network biomarkers (DNB) analysis. The mice used were the same as those in Fig 6. Gene ontology (GO) terms in the category of the Biological process and the related genes are shown in the list. n = 5/group.
(TIF)

S6 Fig GeneChip analysis of hepatic gene expression in olfactory bulbectomized (OBX) mice persistently maintained on a normal chow diet (NCD). C57BL/6J mice (10 weeks old) were subjected to OBX or a sham operation and then maintained on NCD. The liver was isolated 26 weeks after OBX, and a GeneChip/IPA analysis was conducted. Pathway in the category of Molecular and cellular functions and Hepatotoxicity related to changes in hepatic gene expression by OBX are shown in the lists. Molecules mapped to each biological pathway are shown using gene symbols. n = 5/group.
(TIF)

S1 Data Raw data for all figures in this manuscript. (XLSX)

S1 Raw images Representative photographs of the brains obtained from sham-operated (Sham) and olfactory bulbectomized (OBX) mice. (PDF)

## Abbreviations:

DNB: dynamical network biomarkers
eWAT: epididymal white adipose tissue
FOS1: forced olfactory stimulation apparatus with 1 shading bottle
GO: gene ontology
GTT: glucose tolerance test
HFD: high-fat diet
HNF6: hepatocyte nuclear factor 6
i.p.: intraperitoneal
IPA: Ingenuity Pathway Analysis
ITT: insulin tolerance test
iWAT: inguinal white adipose tissue
NEFA: non-esterified fatty acid
NCD: normal chow diet
OBX: olfactory bulbectomized
OFTT: oral fat tolerance test
OGTT: oral glucose tolerance test
*Orexin* ^*-/-*^: orexin knockout
PCR: polymerase chain reaction
RQ: respiratory quotient
RT: room temperature
SEM: standard error of the mean
ZT: zeitgeber time
