# Supplementary figures and images for: Olfactory bulbectomy-induced impairment of lipid utilization leads to abnormal glucose metabolism in mice
Source: PLoS One. 2025 Sep 29;20(9):e0333176. doi: 10.1371/journal.pone.0333176 (PMC12478900; doi:10.1371/journal.pone.0333176)

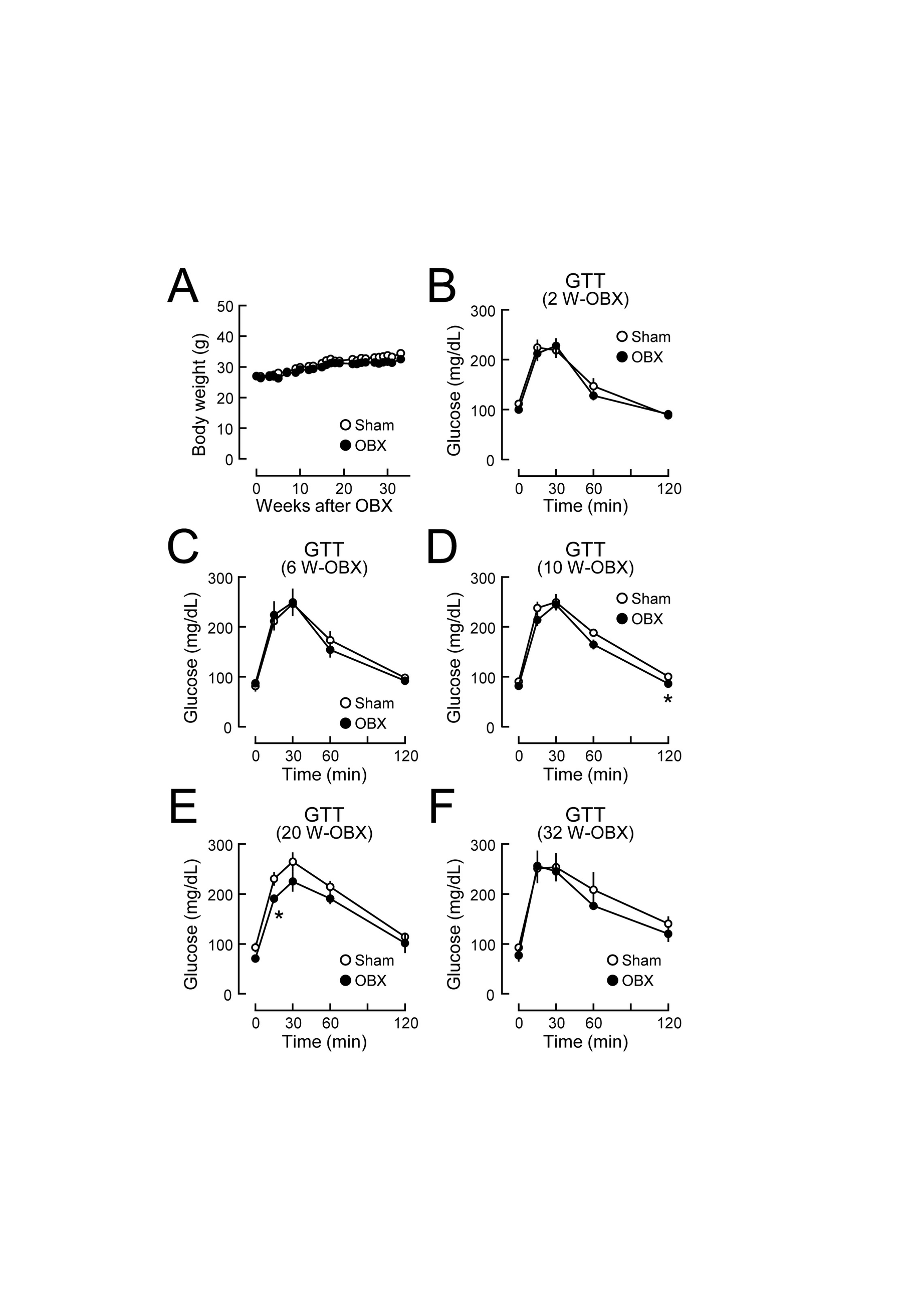

Supplement: S1 Fig — C57BL/6J mice (7 weeks old) were subjected to OBX and maintained on NCD. (A) Similar body weight gain in OBX and sham-operated (Sham) mice. n = 5–6. (B-F) Glucose tolerance tests (GTT) conducted 2 weeks (B), 6 weeks (C), 10 weeks (D), 20 weeks (E), and 32 weeks (F) after OBX. n = 5–6. Values are the means ± SEM. *P < 0.05 assessed by the Student’s t-test. (TIF) [file pone.0333176.s001.tif]

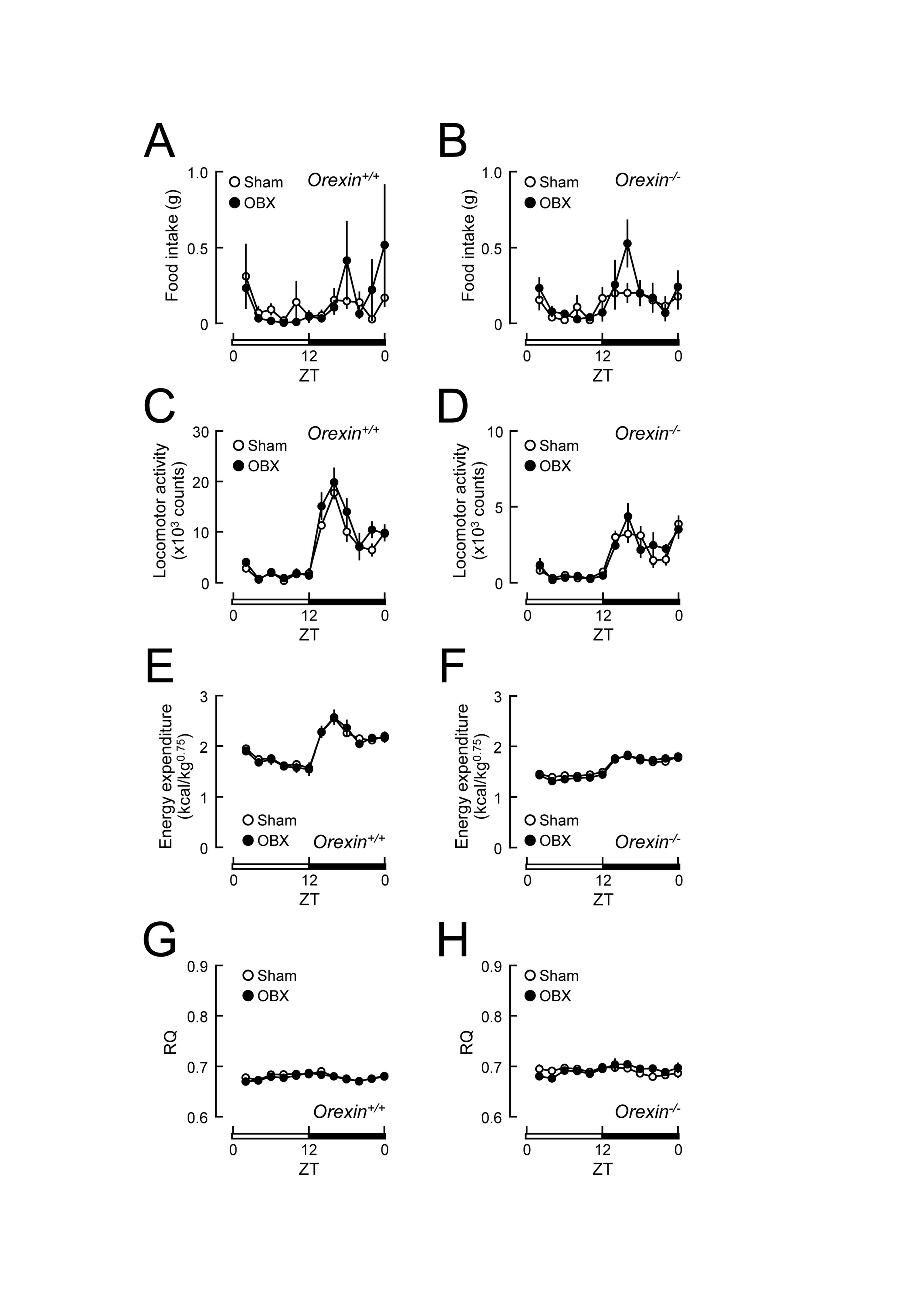

Supplement: S2 Fig — Orexin+/+ and Orexin-/- mice (8–10 weeks old) underwent olfactory bulbectomy, and HFD feeding began 1 week later. A metabolic cage analysis was conducted on mice fed HFD for 24 weeks (i.e., 25 weeks after surgery). (A-B) Food intake in Orexin+/+ mice (A) and Orexin-/- mice (B). n = 5–6. (C-D) Locomotor activity in Orexin+/+ mice (C) and Orexin-/- mice (D). n = 5–6. (E-F) Energy expenditure in Orexin+/+ mice (E) and Orexin-/- mice (F). n = 5–6. (G-H) The respiratory quotient in Orexin+/+ mice (G) and Orexin-/- mice (H). n = 5–6. Values are the means ± SEM. The significance of differences was evaluated by the Student’s t-test. The mice used were the same as those in Fig 4. (TIF) [file pone.0333176.s002.tif]

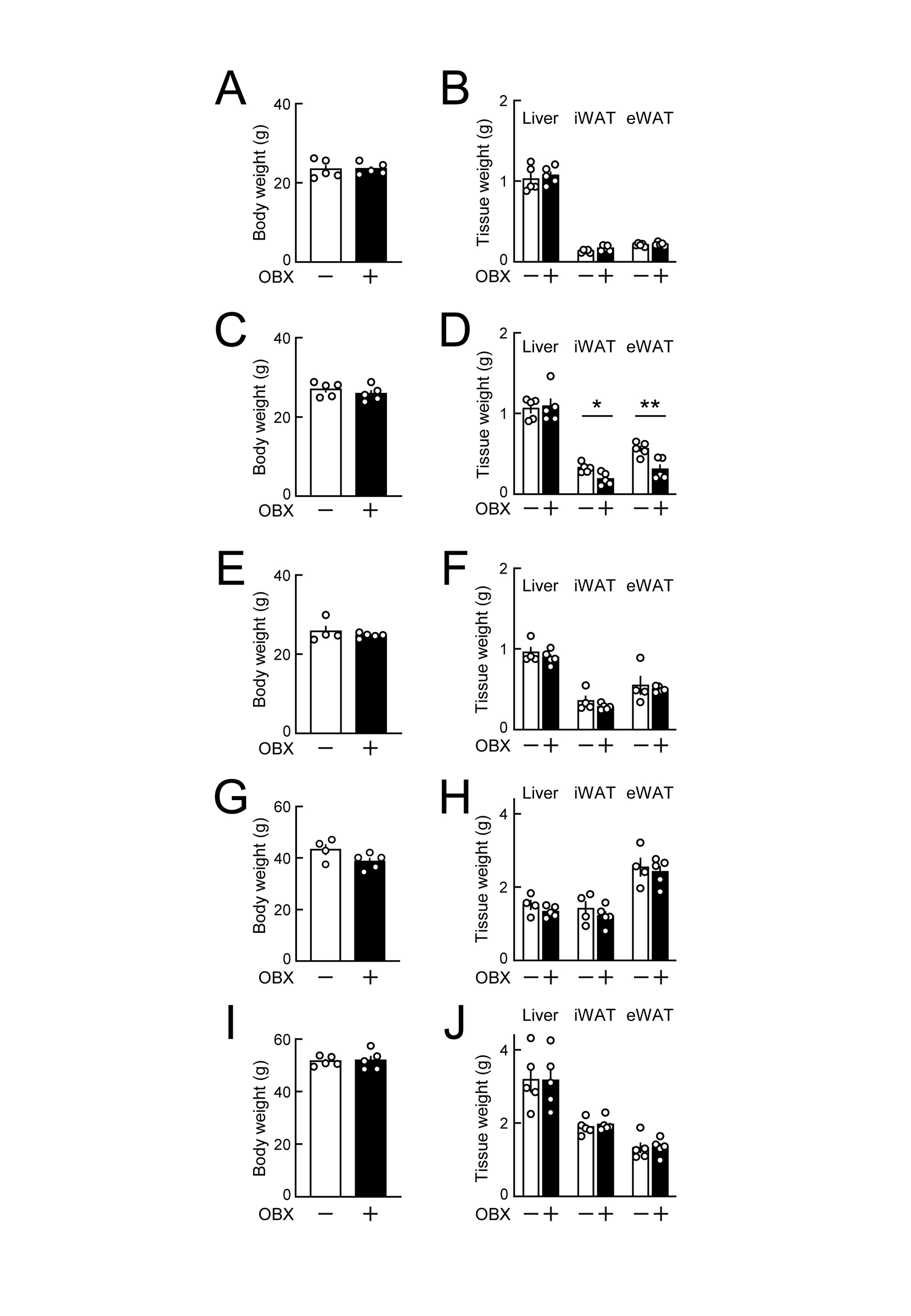

Supplement: S3 Fig — C57BL/6J mice (10 weeks old) underwent olfactory bulbectomy, and HFD feeding began 1 week later. Body weights and weights of peripheral tissues [the liver, inguinal white adipose tissue (iWAT), and epididymal white adipose tissue (eWAT)] in OBX and sham-operated mice sacrificed by cervical dislocation 1 week (A-B), 2 weeks (C-D), 3 weeks (E-F), 10 weeks (G-H), and 26 weeks (I-J) after surgery. n = 4–5/group. The mice used in panels I-J were the same as those in Fig 6. Data are presented as mean ± SEM. *P < 0.05, **P < 0.01 assessed by the Student’s t-test. (TIF) [file pone.0333176.s003.tif]

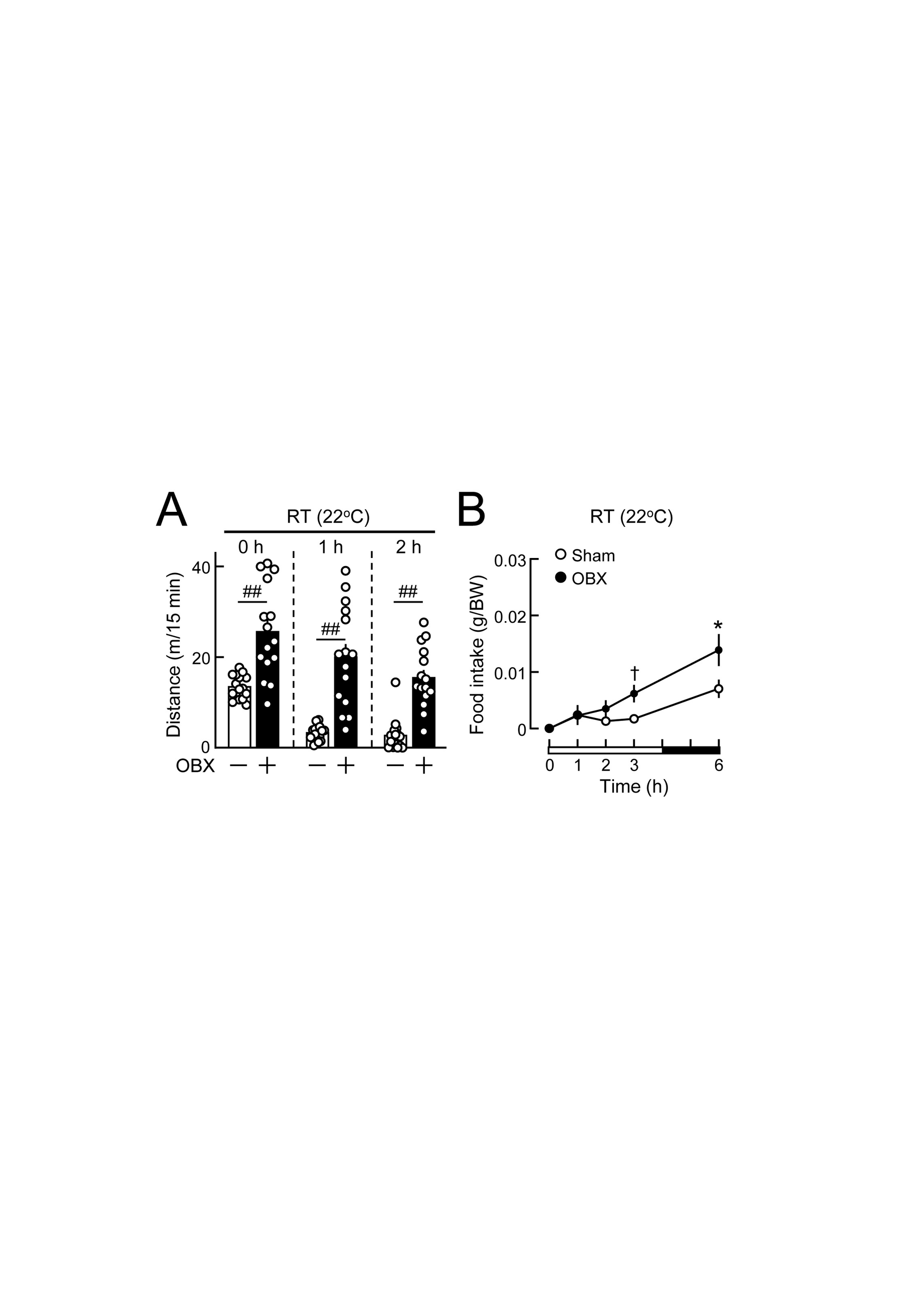

Supplement: S4 Fig — (A) Locomotor activity (total distance) in olfactory bulbectomized (OBX) and sham-operated mice, measured 0 h (0–15 min), 1 h (60–75 min), and 2 h (120–135 min) after a cage change at room temperature (RT, 22oC). Experiments were conducted 49 weeks after surgery. n = 15–16. (B) Cumulative food intake after 6 h of fasting in OBX and sham-operated mice at RT (22oC). Experiments were conducted 48 weeks after surgery. n = 15–16. Values are the means ± SEM. *P < 0.05 assessed by the Student’s t-test. †P < 0.05 assessed by the Welch’s t-test. ##P < 0.01 assessed by the Mann-Whitney U-test. The mice used were the same as those in Fig 2. (TIF) [file pone.0333176.s004.tif]

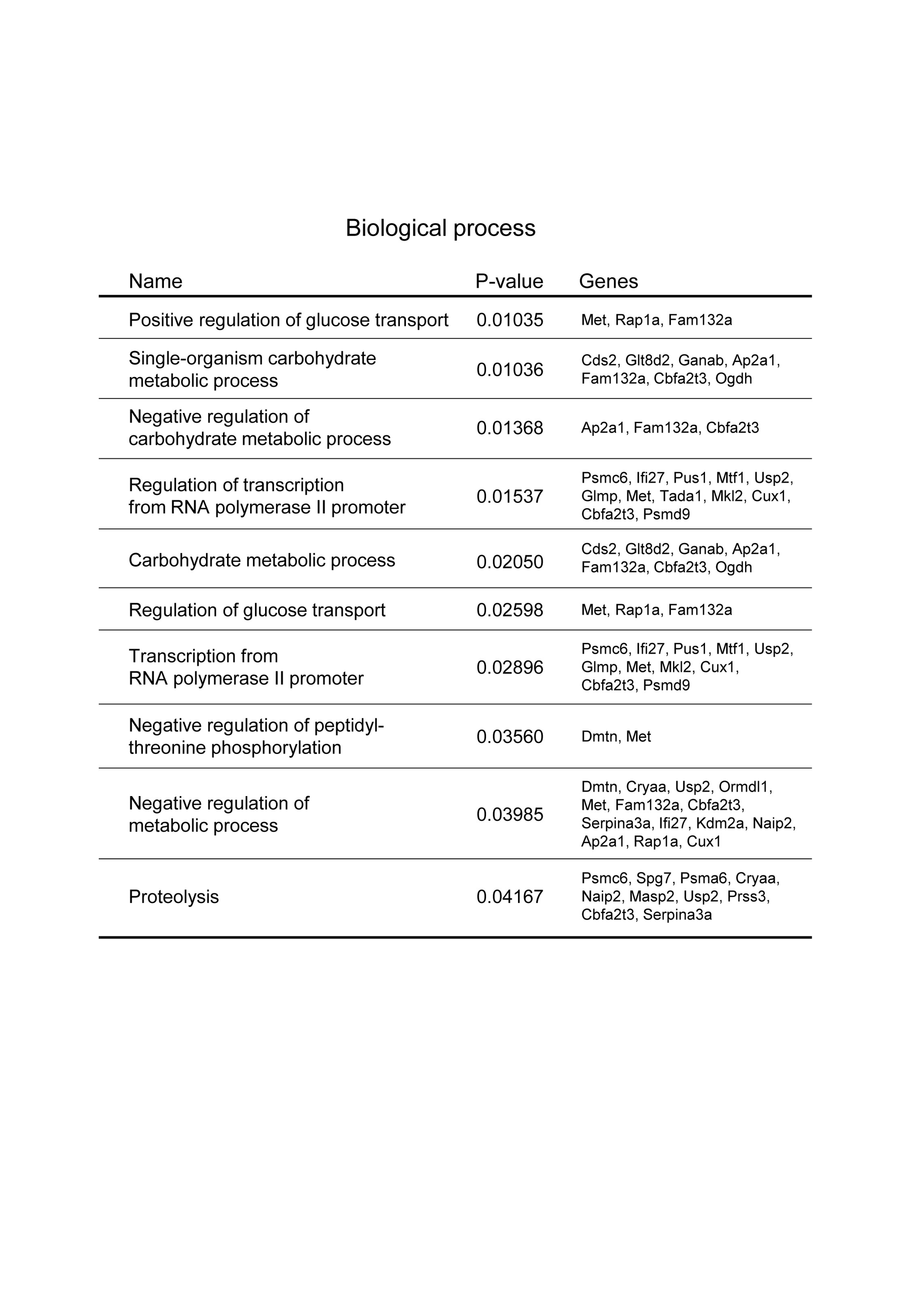

Supplement: S5 Fig — Profiles of hepatic gene expression in OBX mice fed HFD for 25 weeks relative to those in sham-operated mice, as investigated by a dynamical network biomarkers (DNB) analysis. The mice used were the same as those in Fig 6. Gene ontology (GO) terms in the category of the Biological process and the related genes are shown in the list. n = 5/group. (TIF) [file pone.0333176.s005.tif]

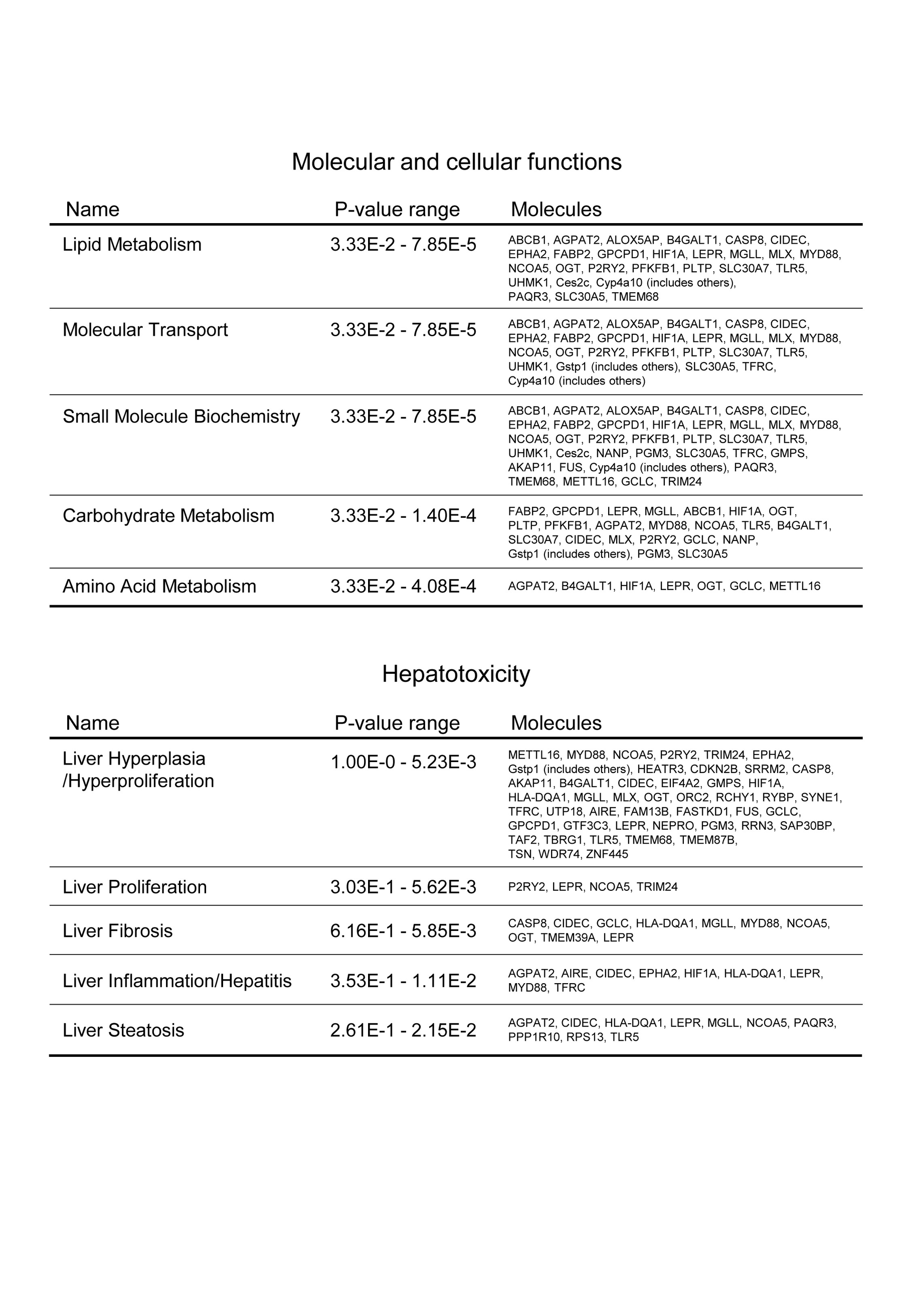

Supplement: S6 Fig — C57BL/6J mice (10 weeks old) were subjected to OBX or a sham operation and then maintained on NCD. The liver was isolated 26 weeks after OBX, and a GeneChip/IPA analysis was conducted. Pathway in the category of Molecular and cellular functions and Hepatotoxicity related to changes in hepatic gene expression by OBX are shown in the lists. Molecules mapped to each biological pathway are shown using gene symbols. n = 5/group. (TIF) [file pone.0333176.s006.tif]

Sham

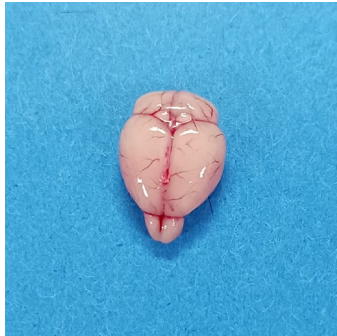

OBX

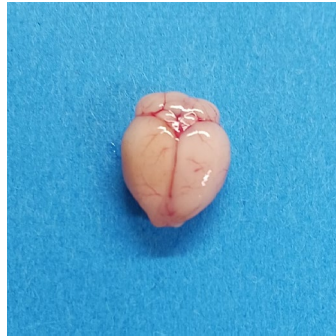

Supplement: S1 Raw images — (PDF) [file pone.0333176.s008.pdf]
